# QuickStats

**Published:** 2014-08-15

**Authors:** FROM THE NATIONAL CENTER FOR HEALTH STATISTICS

**Figure:**
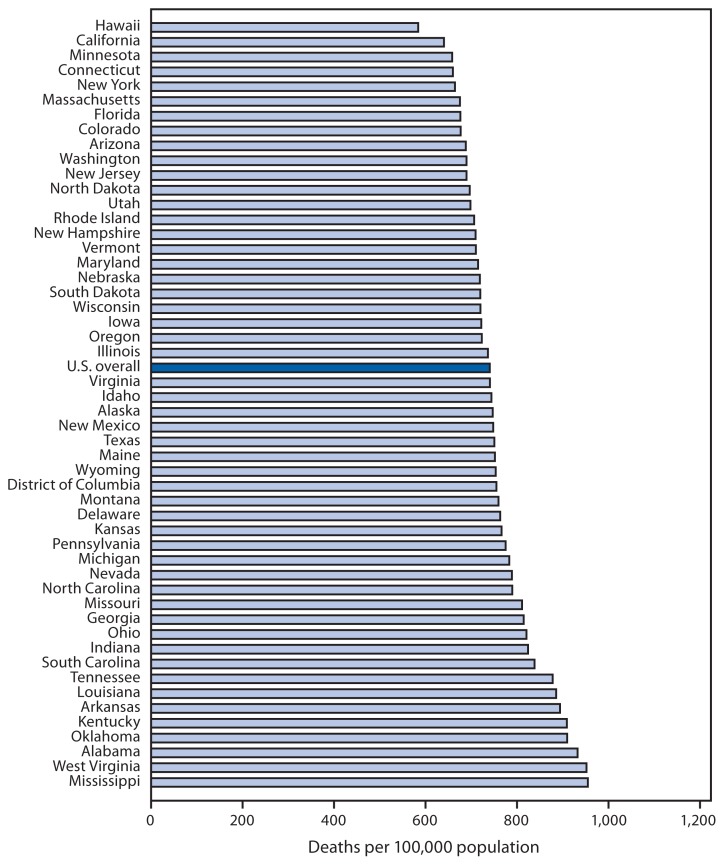
Age-Adjusted Death Rates,* by State^†^ — United States, 2011 * Rates per 100,000 population were calculated based on postcensal populations as of July 1, 2011. ^†^ U.S. residents only.

In 2011, the overall age-adjusted death rate for the United States was 741.3 per 100,000 population. Among states, Mississippi had the highest death rate (956.1), followed by West Virginia (953.2), Alabama (933.6), and Oklahoma (910.9). Hawaii had the lowest death rate (584.9), followed by California (641.3), Minnesota (659.2), and Connecticut (660.6). The rates for 27 states and the District of Columbia were higher than the overall U.S. rate.

**Source:** National Vital Statistics System. Mortality public use data files, 2011. Available at http://www.cdc.gov/nchs/data_access/vitalstatsonline.htm.

**Reported by:** Jiaquan Xu, MD, jax4@cdc.gov, 301-458-4086.

